# Absence of abnormal vascular changes on prenatal imaging aids in differentiating simple uterine scar dehiscence from placenta accreta spectrum: a case series

**DOI:** 10.3389/frph.2023.1068377

**Published:** 2023-10-20

**Authors:** Theophilus K. Adu-Bredu, Yaw Gyanteh Owusu, Atta Owusu-Bempah, Sally L. Collins

**Affiliations:** ^1^ Department of Obstetrics and Gynecology, Komfo Anokye Teaching Hospital, Kumasi, Ghana; ^2^ Nuffield Department of Women’s and Reproductive Health, University of Oxford, Oxford, United Kingdom; ^3^ Fetal Medicine Unit, John Radcliffe Hospital, Oxford, United Kingdom

**Keywords:** uterine window, scar dehiscence, placenta accreta spectrum, uterine scar, prenatal diagnosis, cesarean, maternal morbidity

## Abstract

Accurate prenatal discrimination between a simple, non-adherent uterine scar dehiscence with an underlying placenta and the severe end of the placenta accreta spectrum is problematic as the two can appear similar on prenatal imaging. This may lead to the false diagnosis of placenta accreta spectrum resulting obstetric anxiety, overtreatment and potential iatrogenic morbidity. Despite potential similarities in the etiology, the manifestation and management of these two conditions is very different. The prenatal sonographic features of seven confirmed cases of simple uterine scar dehiscence with an underlying placenta previa were examined. The common sonographic features found for scar dehiscence was a thinned myometrium (<1 mm) overlying a generally homogenous placenta and a placental bulge. There was absence of lacunae and features of hypervascularity including bridging vessels. Our findings suggest accurate discrimination between a simple scar dehiscence with the placenta underlying it and placenta accreta spectrum can be made on prenatal ultrasound if the placenta is carefully examined for the vascular features unique to PAS.

## 1. Introduction

Simple uterine scar dehiscence with the placenta lying beneath the defect is often confused with placenta accreta spectrum (PAS) both on prenatal imaging and intraoperatively (1). Both conditions result from previous uterine scarring and may even have a pathophysiological overlap, however, as the degree of placental attachment is very different, the surgical management of each should also be different. In instances of simple uterine scar dehiscence, while it may be necessary to remove the overlying serosa layer that covers the placenta bed, the rest of the placenta can be readily detached from the myometrium surrounding the defect. Moreover, there is usually sufficient residual uterine tissue allowing for preservation of the uterus in the majority of cases. Hysterectomy is only performed when the myometrium surrounding the defect fails to adequately contract (usually when the defect is very close to the cervix). Although a definitive diagnosis of PAS can only be made at delivery when the placenta fails to detach, the current management approach discourages any attempt to manually remove the placenta due to the risk of severe hemorrhage which may lead to mortality or severe morbidity (2). Hence, caesarean hysterectomy with the placenta in-situ is often considered to be the gold standard treatment for PAS cases (3).

Undiagnosed PAS is undeniably every obstetrician's nightmare, therefore prenatal diagnosis plays an integral role in influencing the decision for a caesarean hysterectomy and other aggressive management approaches, so obstetricians are at a high risk of confirmation bias (4). This may lead to an overly aggressive management approach including vertical abdominal incision, use of ureteric stents, interventional radiology, and a potentially unnecessary hysterectomy. All of these interventions also increase the risk of iatrogenic morbidity. Prenatal ultrasound assessment plays a critical role in the pre-operative planning and management of both simple scar dehiscence and PAS. Despite its importance, accurate prenatal discrimination between these two closely related but clinically different conditions is challenging. We aimed to investigate the ultrasound features observed in confirmed cases of simple scar dehiscence with underlying placenta and to evaluate which of the standardized ultrasound descriptors (5) may be used to distinguish it from PAS.

## 2. Materials and methods

We included all patients who had intraoperative diagnosis of simple uterine dehiscence with an underlying placenta in the Obstetrics and Gynecology Department of Komfo Anokye Teaching Hospital, Kumasi, Ghana between December 2020 and July 2022. The study had local ethical approval (KATH IRB/AP/111/22).

All the women had an anterior low lying or previa placenta and a history of at least one previous caesarean delivery so were considered to be at high risk of PAS. The placenta was regarded as low lying when the lower edge of the placenta was less than 20 mm from the internal os and previa when it covered the internal os (6). The women underwent a detailed transabdominal and transvaginal ultrasound assessment within one-week prior to surgery which included assessment with color Doppler by a sonographer experienced in the diagnosis of PAS, using Siemens NX 3 Ellite or Samsung SonoAce x7 ultrasound machine. All examinations were performed with a moderately filled urinary bladder and all the signs of PAS defined by the European Working Group on Abnormally Invasive Placenta(EW-AIP) (5) were actively sought.

Data relevant to the pregnancy were extracted from the hospital notes including age, parity, previous uterine surgery, gestational age at delivery, placental location, presence of antepartum hemorrhage. The ultrasound report, images and intraoperative images were retrieved for analysis. Intraoperative information such as surgical outcome (hysterectomy vs. preservation of uterus), estimated blood loss, iatrogenic injury of surrounding viscera, need for blood transfusion were also collected. Only women who were confirmed at delivery as having a simple uterine dehiscence with a placenta which was easily separated from the surrounding myometrium were included.

## 3. Results

Seven women had a uterine scar dehiscence overlying the placenta during the study period. The patient characteristics, ultrasound and intraoperative findings are reported in Table 1. All the women had had at least one caesarean delivery, with five having had two and one having had three. In all cases, the placenta was implanted at the presumed site of the previous caesarean section scar. Three of the women had experienced at least one episode of antepartum hemorrhage.

**Table 1:** Showing patient characteristics, ultrasound and intraoperative findings.

Case	Gestational age at delivery	No. of previous c-section	Ultrasound findings	PAI score [probability of invasion (%)]	Intraoperative findings	Management	Estimated blood loss
Case 1	33 ^+ 2^ weeks	1	• Myometrial thinning of 0.6 mm overlying the anterior low placenta • Placenta bulge towards bladder • Fairly homogenous placenta • Placental lakes with no feeder vessels confirmed with color Doppler • Retroplacental hypoechoic collection	• Score = 2 • Probability of invasion = 10%	• Thinned lower uterine segment with contents visible (only serosa covering the placenta and retroplacental clot) • Huge placental bulge. • Normal appearing myometrial tissue on uterine surface.	• Transverse uterine incision • Placental separation with no difficulty. • Uterus was preserved • No massive placenta bed hemorrhage • No blood transfusion	700 mls
Case 2	36 ^+ 0^ weeks	2	• Myometrial thinning of 0.7 mm overlying the anterior low placenta • Fairly homogenous placenta • Placental lakes with no feeder vessels confirmed with color Doppler	• Score = 5 • Probability of invasion = 51%	• Thinned lower uterine segment with only serosa covering the placenta spanning at length of 4 cm vertically. • Normal appearance of myometrial tissue on uterine surface	• Transverse uterine incision • Placental separation with no difficulty. • Uterus was preserved • No massive placental bed hemorrhage • No blood transfusion	1,100 mls
Case 3	36 ^+ 1^ weeks	3	• Imperceptible myometrium overlying the anterior low placenta • Placental bulge towards bladder • Fairly homogenous placenta • Placental lakes with no feeder vessels confirmed with color Doppler	• Score = 5 • Probability of invasion = 51%	• Thinned lower uterine segment with only serosa overlying the visible placenta. • Huge placental bulge • Normal appearance of surrounding myometrial tissue on uterine surface	• Classical uterine incision. • Placental separation with minimal resistance. • Significant placental bed hemorrhage mostly from the cervical area. • Hysterectomy due to difficulty to control hemostasis • 2 units of whole blood given	1,700 ml
Case 4	36 ^+ 0^ weeks	2	• Imperceptible myometrial tissue overlying the anterior low placenta. • Fairly homogenous placenta • Placental lakes with no feeder vessels confirmed with color Doppler	• Score = 5 • Probability of invasion = 51%	• Thinned lower uterine segment with the serosa overlying the partially visible placenta. • Normal appearance of the surrounding myometrial tissue	• Transverse uterine incision • Placental separation with no difficulty • Uterus was preserved • No massive placental bed hemorrhage • No blood transfusion	700 ml
Case 5	37 ^+ 0^ weeks	2	• Myometrial tissue at LUTS showing some areas with imperceptible myometrium and other areas showing normal myometrial thickness of about 2.6 mm • A fairly homogenous placenta • Placental lakes with no feeder vessels confirmed with color Doppler	• Score = 5 • Probability of invasion = 51%	• Punctate areas of scar dehiscence with the placenta bulging through, interspersed within areas of normal myometrium at the lower uterine segment. • Normal appearance of the surrounding myometrium.	• Transverse uterine incision • Placenta separation with no difficulty • Uterus was preserved • No massive placental bed hemorrhage • No blood transfusion	800 ml
Case 6	36 ^+ 0^ weeks	2	• Myometrial thinning about 0.6 mm overlying the anterior low placenta. • A fairly homogenous placenta with some areas of calcifications. • Placental lakes with no feeder vessels confirmed with color Doppler	• Score = 5 • Probability of invasion = 51%	• Intraoperative findings revealed a completely dehisced uterine scar with the placenta clearly visualized through. • Normal appearance of the surrounding myometrium	• Transverse uterine incision • Placenta separation with no difficulty • Uterus was preserved • No massive placental bed hemorrhage • No blood transfusion	700 ml
Case 7	35 ^+ 0^ weeks	2	• Imperceptible myometrium overlying the anterior low placenta. • A fairly homogenous placenta • Placental lakes with no feeder vessels confirmed with color Doppler	• Score = 5 • Probability of invasion = 51%	• A large uterine scar dehiscence in the • lower uterine segment spanning a length of 6 cm with the placenta partly bulging through the defect • Normal appearance of the surrounding myometrium	• Transverse uterine incision • Placenta separation with no difficulty • Uterus was preserved • No massive placental bed hemorrhage • No blood transfusion	1,100 ml

**Figure 1:**
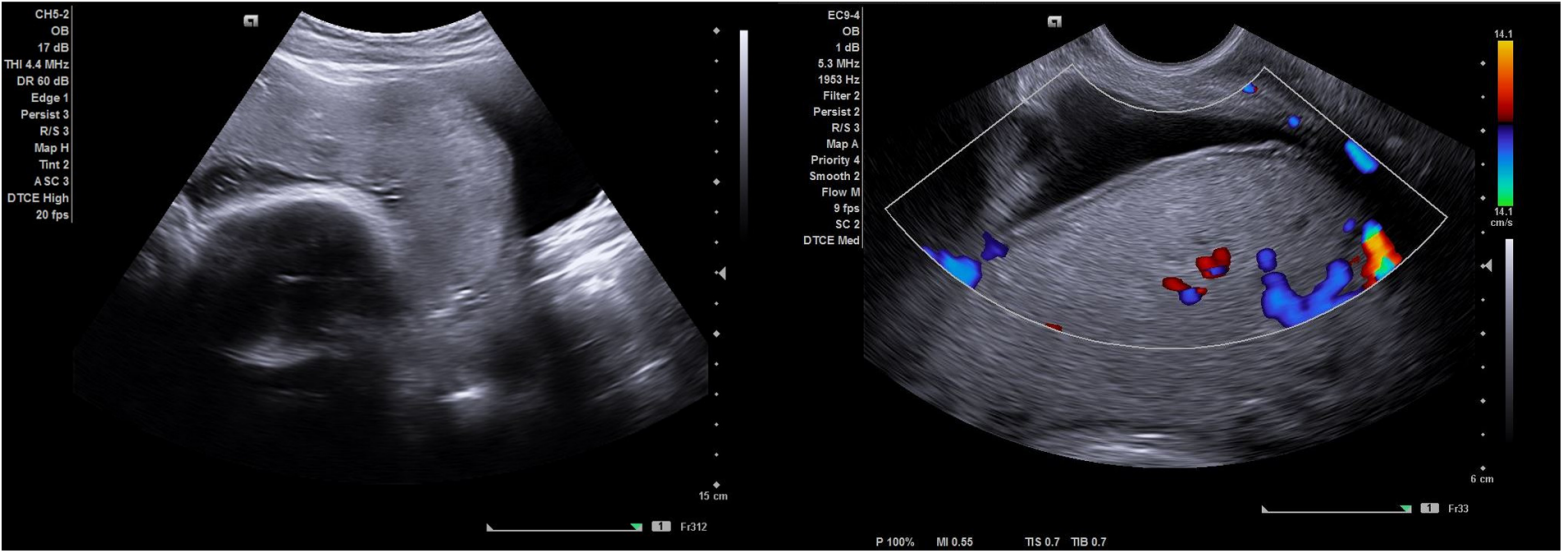
Shows an ultrasound image of a simple scar dehiscence overlying the placenta. Notice the absence of retroplacental hypervascularity, bridging vessels and abnormal lacunae (with no feeder vessels) observed on gray scale imaging and color Doppler.

On ultrasound examination abnormally thin myometrium (<1 mm) was observed to be overlying the placenta in all seven women. All the placentas were homogenous with a few simple lakes which were regular in shape and had no high velocity feeder vessels. One placenta had peripheral calcification which is usually seen in ageing placentas. An obvious placental bulge i.e., a deviation of the uterine contour at the lower uterine segment towards the urinary bladder, was seen in two of the seven women. No other EW-AIP ultrasound signs of PAS were seen (Figure 1).

Caesarean delivery was performed through the previous transverse abdominal incision in six cases. A classical incision was used for the seventh case due to a high index of suspicion for PAS from an ultrasound examination performed early in pregnancy. In all cases, intraoperative findings revealed at least one area of uterine dehiscence where the placenta was directly visible under a layer of serosa. No evidence of neovascularization was seen on the serosal surface or in the utero-vesical fold of peritoneum between the uterus and bladder in any of the seven cases. The placenta spontaneously separated from the myometrium surrounding the defect in six women, controlled cord traction was required for the seventh. Excessive bleeding after the placental delivery occurred in case 3. As there was a large dehiscence very close to the cervix the bleeding was difficult to control and a hysterectomy was performed. Estimated blood loss was less than 1,100 ml for all the women except case 3 who had the hysterectomy. She lost 1,700 ml. Only case 3 required a blood transfusion, she received 2 units of whole blood during the hysterectomy.

Delivery was between 36- and 37-weeks' gestation for five cases. Early delivery occurred in two cases due to clinical concerns. In case 1, the woman presented with symptoms of concealed placental abruption and so was delivered as an emergency. A large retroplacental clot was found at delivery appearing to confirm the clinical suspicion. In case 7, the woman was delivered as an emergency due to severe lower abdominal pain which was suspected to be uterine rupture. No rupture was found at laparotomy.

There were no maternal mortalities, visceral injuries, or postnatal complications. All the mothers were discharged home three days postnatally. There was a positive outcome for all the babies with no morbidity or mortality. The maximum admission to the neonatal intensive care unit (NICU) was three days.

## 4. Discussion

The common sonographic findings of simple uterine scar dehiscence in these cases were significant myometrial thinning (<1 mm or vanishingly thin), homogenous placenta and a placental bulge (Table 1). Intraoperative findings were a thin lower uterine segment with the placenta seen directly underneath, absence of neovascularity on the serosa surface and normal surrounding myometrial tissue. The placenta completely separated in all cases and the uterus was preserved in all but one woman who required a hysterectomy as a result of bleeding from the cervix. This suggests that accurate discrimination between PAS and simple uterine scar dehiscence with an underlying placenta may be possible with prenatal ultrasound by carefully evaluating the uteroplacental bed for markers of neovascularity which are not present in simple dehiscence.

**Figure 2:**
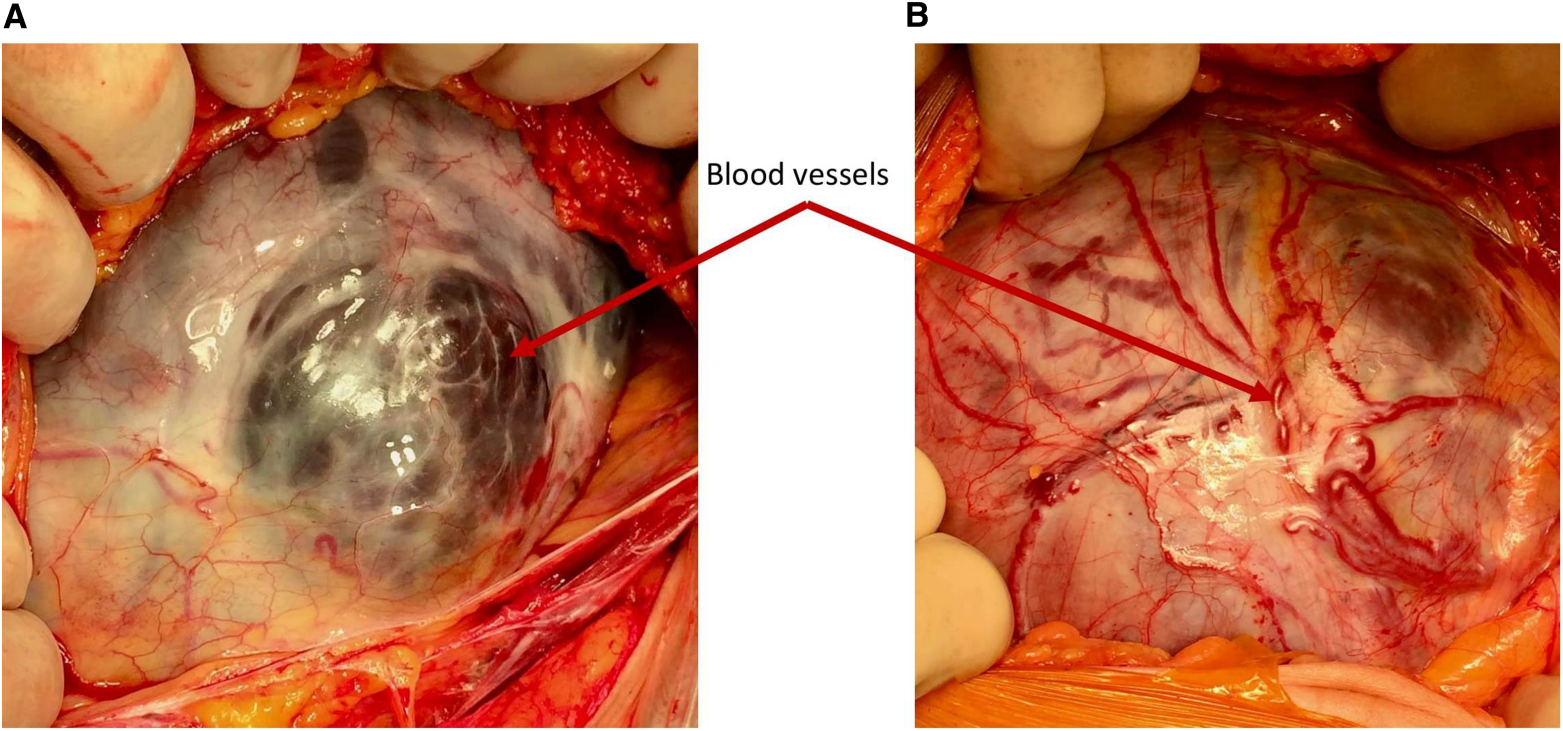
Demonstrating the difference in the vascularity. (**A**) Simple dehiscence—the blood vessels seen through the defect are large, dark and situated under the serosa surface (**B**) PAS—the blood vessels over the placental bed are bright red and running in or over the serosa and are often seen passing cranio-caudally.

Remodeling of the lower uterine segment in pregnancies after a previous caesarean delivery is a common phenomenon and may be explained by poor myometrial healing (7, 8) with reduction of the myofibres and muscle density at the site of the scar in the lower uterine segment (9). This phenomenon results in an increased risk of progressive scar dehiscence, uterine rupture and PAS in subsequent pregnancies. However, when an area of scar dehiscence overlies the placenta, it may be confused with PAS. Intraoperatively, a differentiation can be made due to the absence of neovascularization on the serosa surface and completely normal myometrium surrounding the usually regular scar defect (2). It must be noted that large placental vessels may be seen running underneath the serosa in a simple dehiscence, however it is the presence of neovascularity on the surface of the serosa that indicates PAS (see Figure 2). On the other hand, prenatal diagnosis is often not straightforward since scar dehiscence can occur alone or in combination with PAS. In view of this, a thorough ultrasound examination of the placental bed should be performed and the uterus carefully inspected at laparotomy before deciding between the two diagnoses.

In an attempt to improve prenatal diagnosis of PAS, placental accreta index score (PAI) was developed (10). However, this scoring system is poor at differentiating between cases of PAS and scar dehiscence with underlying placenta. Applying the PAI scoring system to this case series would predict a probability of abnormal invasion above 50% in 6 out of the 7 cases (Table 1), this is potentially enough evidence for the surgeon to proceed with a caesarean hysterectomy particularly after visualizing the placenta directly beneath the serosa. Similarly, the proposed imaging descriptors proposed by EW-AIP (now International Society of Placenta Accreta Spectrum) (5) do not provide guidance on distinguishing between these two conditions.

Traditionally, PAS has been attributed to the invasion of the extravillous trophoblast (EVT) through defective decidua resulting in the direct attachment or invasion of the villi through the myometrium (11). Recently, it has been suggested that PAS results from the implantation of EVT at the area of defective decidua with progressive scar dehiscence as the placenta grows (12, 13). This theory also refutes the existence of placenta percreta and attributes any breach seen in the uterine serosa to uterine rupture as a result of surgical manipulation and dissection (11). It also attributes surrounding visceral structures involvement to pelvic adhesive disease (14). In an era of intensive research into PAS, this recent theory may potentially affect the management approach of this condition. However, a major issue with this is the possible confusion among clinicians between a placenta underlying a simple scar dehiscence and PAS.

On ultrasound the placenta appears homogenous with the absence of abnormal vascular changes (1). On the other hand, the villous penetration of the myometrium in PAS reaches the deep myometrial vessels such as the radial and arcuate arteries which results in the formation of abnormally large vascular channels within the placenta (lacunae). Also seen are large amounts of neovascularisation on the serosal surface and in the utero-vesical fold (15). On prenatal ultrasound, this abnormal vascularity should be readily seen with color Doppler (16). Placental lakes and echogenic cystic lesions, are normal morphological findings that become more prominent in ageing placentas. These should be differentiated from abnormal PAS lacunae by their much greater size, irregularity, absence of high-flow feeder vessels on color Doppler and where it is possible to demonstrate, easy compressibility (17). Placental lakes and infarcts may be seen in non-PAS placentas and must not be confused with the abnormal lacunae seen in PAS (16). Also, urinary bladder varicosities (which is a common finding in pregnancy) found at the uterovesical interface can be differentiated from neovascularity by their characteristic low velocity, parallel course to the urinary bladder wall and extension away from the uterovesical interface (18).

This case series has several limitations. Firstly, the sample size is small. This is inevitable as the incidence of uterine dehiscence overlying a placenta previa is low. However, as all the cases demonstrated the same ultrasound signs with no markers of neovascularity, it is appropriate to suggest that this should be considered by sonographers when recording their antenatal suspicions. This is a prospectively collected case series with no PAS case-matched controls. This said, the EW-AIP standardized markers are well described and the underlying aim was to assess the presence of these markers in placentas which were not PAS but simple dehiscence. Finally, all ultrasound studies were undertaken by a single operator and so the intra-operator variability remains unknown.

This case series seeks to draw the attention of obstetricians and sonographers to the differential diagnosis of simple scar dehiscence with an underlying placenta which occurs in pregnancies at risk of PAS. Discriminating between these two closely related conditions on prenatal imaging is not straightforward as it requires experience and in-depth knowledge of the pathophysiology and how that relates to the ultrasound findings. However, accurate prenatal diagnosis is essential for planning delivery as the intraoperative management and multidisciplinary approach differs significantly between the two. Also, there may be a higher risk of uterine rupture with a simple scar dehiscence hence increased observation of the patient and earlier delivery may be necessary.

## 5. Conclusion

Accurate discrimination between simple uterine scar dehiscence and PAS is possible on prenatal ultrasound. Scar dehiscence manifests as a thinned myometrium overlying a generally homogenous placenta often with a placental bulge, no PAS lacunae with high flow feeder vessels are seen and there are no signs of hypervascularity, notably no bridging vessels. Referral for a second opinion from a PAS expert is always to be encouraged. One of the most useful ways of differentiating between the two pathologies at laparotomy is to evaluate the uterine surface intraoperatively. In simple dehiscence, there should not be large vessels on the surface of the serosa (neovascularity) although vessels are often seen beneath the serosal surface and the myometrium surrounding the defect should be normal and regular in appearance. If there is any doubt, the uterine incision should be placed away from the placenta and diagnosis made according to separation or lack of it after delivery of the baby.

## Data Availability

The raw data supporting the conclusions of this article will be made available by the authors, without undue reservation.

## References

[1] Adu-Bredu TK Owusu-Bempah A Collins S. Accurate prenatal discrimination of placenta accreta spectrum from uterine dehiscence is necessary to ensure optimal management. BMJ Case Rep. (2021) 14:e244286. 10.1136/bcr-2021-244286 34244192 PMC8273451

[2] Collins SL Alemdar B van Beekhuizen HJ Bertholdt C Braun T Calda P Evidence-based guidelines for the management of abnormally invasive placenta: recommendations from the international society for abnormally invasive placenta. Am J Obstet Gynecol. (2019) 220:511–26. 10.1016/j.ajog.2019.02.054 30849356

[3] Di Mascio D Calì G D’antonio F. Updates on the management of placenta accreta spectrum. Minerva Ginecol. (2019) 71:113–20. 10.23736/S0026-4784.18.04333-2 30486635

[4] Jauniaux E Hussein AM Einerson BD Silver RM. Debunking 20th century myths and legends about the diagnosis of placenta accreta spectrum. Ultrasound Obstet Gynecol. (2022) 59:417–23. 10.1002/uog.24890 35363412

[5] Collins SL Ashcroft A Braun T Calda P Langhoff-Roos J Morel O Proposal for standardized ultrasound descriptors of abnormally invasive placenta (AIP). Ultrasound Obstet Gynecol. (2016) 47:271–5. 10.1002/uog.14952 26205041

[6] Reddy UM Abuhamad AZ Levine D Saade GR. Participants for the FIWI. Fetal imaging. J Ultrasound Med. (2014) 33:745–57. 10.7863/ultra.33.5.745 24764329

[7] Roeder HA Cramer SF Leppert PC. A look at uterine wound healing through a histopathological study of uterine scars. Reprod Sci. (2012) 19:463–73. 10.1177/1933719111426603 22344737

[8] Wu C Chen X Mei Z Zhou J Wu L Chiu W-H A preliminary study of uterine scar tissue following cesarean section. J Perinat Med. (2018) 46:379–86. 10.1515/jpm-2016-0347 28961140

[9] Schwalm H Dubrauszky V. The structure of the musculature of the human uterus—muscles and connective tissue. Am J Obstet Gynecol. (1966) 94:391–404. 10.1016/0002-9378(66)90661-2 5905056

[10] Rac MWF Dashe JS Wells CE Moschos E McIntire DD Twickler DM. Ultrasound predictors of placental invasion: the placenta accreta index. Am J Obstet Gynecol. (2015) 212:343.e1–e7. 10.1016/j.ajog.2014.10.022 25446658

[11] Jauniaux E Jurkovic D. Placenta accreta: pathogenesis of a 20th century iatrogenic uterine disease. Placenta. (2012) 33:244–51. 10.1016/j.placenta.2011.11.010 22284667

[12] Jauniaux E Hussein AM Elbarmelgy RM Elbarmelgy RA Burton GJ. Failure of placental detachment in accreta placentation is associated with excessive fibrinoid deposition at the utero-placental interface. Am J Obstet Gynecol. (2022) 226:243.e1–243.e10. 10.1016/j.ajog.2021.08.026 34461077

[13] Einerson BD Comstock J Silver RM Branch DW Woodward PJ Kennedy A. Placenta accreta spectrum disorder: uterine dehiscence, not placental invasion. Obstet Gynecol. (2020) 135:1104–11. 10.1097/AOG.0000000000003793 32282597

[14] Jauniaux E Hecht JL Elbarmelgy RA Elbarmelgy RM Thabet MM Hussein AM. Searching for placenta percreta: a prospective cohort and systematic review of case reports. Am J Obstet Gynecol. (2022) 226:837.e1–837.e13. 10.1016/j.ajog.2021.12.030 34973177

[15] D’Antonio F Iacovella C Bhide A. Prenatal identification of invasive placentation using ultrasound: systematic review and meta-analysis. Ultrasound Obstet Gynecol. (2013) 42:509–17. 10.1002/uog.13194 23943408

[16] Adu-Bredu TK Rijken MJ Nieto-Calvache AJ Stefanovic V Aryananda RA Fox KA A simple guide to ultrasound screening for placenta accreta spectrum for improving detection and optimizing management in resource limited settings. Int J Gynaecol Obstet. (2023) 160:732–41. 10.1002/ijgo.14376 35900178 PMC10086861

[17] Jauniaux E Collins S Burton GJ. Placenta accreta spectrum: pathophysiology and evidence-based anatomy for prenatal ultrasound imaging. Am J Obstet Gynecol. (2018) 218:75–87. 10.1016/j.ajog.2017.05.067 28599899

[18] Adu-Bredu TK Collins SL Nieto-Calvache AJ. Ultrasound discrimination between placenta accreta spectrum and urinary bladder varices. Aust N Z J Obstet Gynaecol. (2023) in press. 10.1111/ajo.13703 37872717

